# Packing polymorphism in the structure of *trans*-aqua­[*N*,*N*′-bis­(salicyl­idene)ethane-1,2-di­amine-κ^4^ *O*,*N*,*N*′,*O*′]chlorido­manganese(III) monohydrate

**DOI:** 10.1107/S2056989018015694

**Published:** 2018-11-13

**Authors:** Juan Alberto Reyes Perea, Sylvain Bernès, Ma Guadalupe Quintero Téllez

**Affiliations:** a Facultad de Ciencias Físico Matemáticas, Benemérita Universidad Autónoma de Puebla, 72570 Puebla, Pue., Mexico; b Instituto de Física, Benemérita Universidad Autónoma de Puebla, 72570 Puebla, Pue., Mexico; c Facultad de Ciencias Químicas, Benemérita Universidad Autónoma de Puebla, 72570 Puebla, Pue., Mexico

**Keywords:** crystal structure, polymorphism, salen ligand, manganese, hydrate

## Abstract

The crystal structure of the title complex (systematic name: *trans*-aqua­chlorido­{2,2′[ethane-1,2-diylbis(nitrilo­methyl­idyne)]diphenolato-κ^4^ *O*,*N*,*N*′,*O*′}manganese(III) monohydrate), [Mn(C_16_H_14_N_2_O_2_)Cl(H_2_O)]·H_2_O has been reported previously in the space group *P*2_1_/*n* [Panja *et al.* (2003 ▸). *Polyhedron*, **22**, 1191–1198]. We obtained the same hydrated complex through an alternative synthesis, and crystallized a new polymorph, in the space group *P*2_1_. The mol­ecular conformation of the complex is virtually unmodified, but the absence of the glide plane in the new polymorph halves the unit-cell parameter *c*, affording a non-centrosymmetric crystal structure with *Z* = 2, while the previously reported crystal is centrosymmetric with *Z* = 4. Both phases represent a case of packing polymorphism, similar to other dimorphic crystal structures retrieved from the Cambridge Structural Database.

## Abstract 2

The crystal structure of a second phase, which results from packing polymorphism, is described for a previously reported Mn^III^ complex.

## Chemical context   

Schiff base organic compounds are widely employed ligands in modern coordination chemistry because they are easily accessible and display high versatility (Zarei *et al.*, 2015 ▸). Within this field, the coordination chemistry of H_2_ *salen* [*N*,*N*′-bis­(salicyl­idene)ethane-1,2-di­amine] has been studied with virtually all transition metals. The chelating character of the dianionic ligand *salen* is known to stabilize not only *M* ^2+^ cations, but also higher oxidation states, providing that ancillary anions such as Cl^−^ are present. In the case of manganese, this strategy may be used to stabilize Mn^III^ and Mn^IV^ oxidation states, generally in octa­hedral ligand fields. The resulting complexes are potentially of inter­est in various inter­disciplinary fields such as structural chemistry, catalytic processes involving metalloproteins or enzymes (Sarkar *et al.*, 2017 ▸), magnetochemistry (Blinov, 2017 ▸), and NLO materials. Regarding the sub-family of Mn^III^–s*alen* derivatives, they have been used mainly as models for biological systems involving this metal cation. For our part, we focus on s*alen*-based materials, which can display non-linear optical response, for example with Co^III^ as the metal centre (Qu­intero-Téllez *et al.*, 2016 ▸).

While extending our work to Mn^III^, we prepared the title complex, for which a synthesis was previously reported (Panja *et al.*, 2003 ▸). These authors synthesized the complex using a Mn^III^ compound as starting material, namely [Mn(*salen*)OAc]·H_2_O, which was reacted with MnCl_2_·4H_2_O in water. Crystallization at room temperature afforded brownish black microcrystals, and the authors characterized the complex in space group *P*2_1_/*n*, with *Z* = 4. We obtained the same compound through a more straightforward synthetic route, using a one-pot reaction between salicyl­aldehyde, di­ethyl­enetri­amine, and MnCl_2_, in MeOH. In contrast to the previous synthesis, crystallization was carried out at low temperature (283 K) in methanol, affording brown crystals. The structure determination shows that this phase crystallizes in space group *P*2_1_, with *Z* = 2.
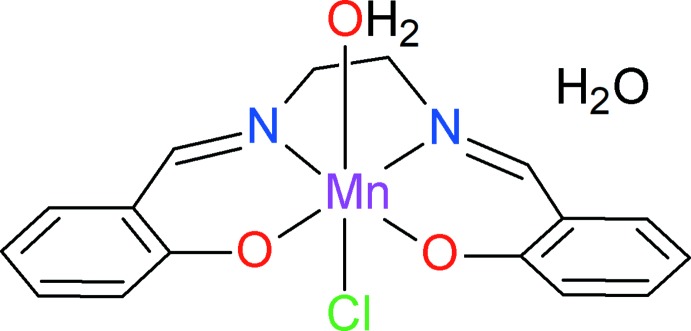


Although we have no strong experimental evidences regarding the mechanism triggering the polymorphism for this complex, we believe that the temperature and the solvent of crystallization could be the key parameters. We report here the structure of the *P*2_1_ polymorph, along with its characterization in solution by means of UV–Vis spectroscopy.

## Structural commentary   

**Figure 1:**
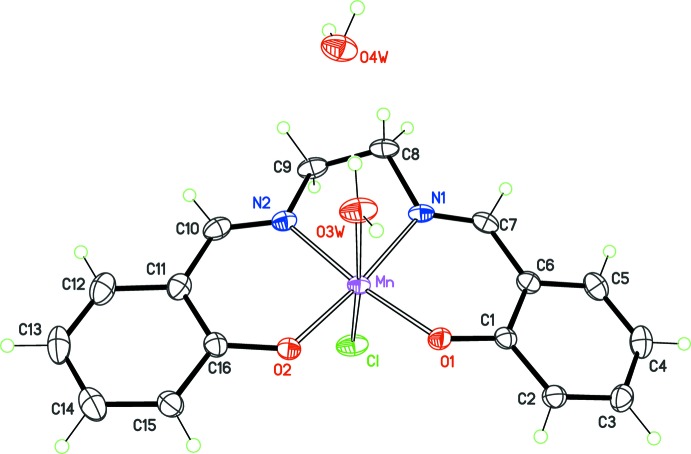
The structure of the title solvate, with displacement ellipsoids for non-H atoms at the 50% probability level.

The asymmetric unit of the *P*2_1_ phase contains one [Mn(*salen*)(OH_2_)Cl] neutral complex and one lattice water mol­ecule, both in general positions (Fig. 1 ▸). As expected, the Mn^III^ centre displays a slightly distorted octa­hedral geometry, with the four donor sites of ligand *salen* in the equatorial plane (N1/N2/O1/O2). The metal deviates by only 0.056 Å from the equatorial plane, and axial sites are occupied by a water mol­ecule (O3*W*) and the chloride ion (Cl) at normal distances. Deviations from an ideal octa­hedral geometry result from the bite angles of the chelating *salen* ligand.

**Figure 2:**
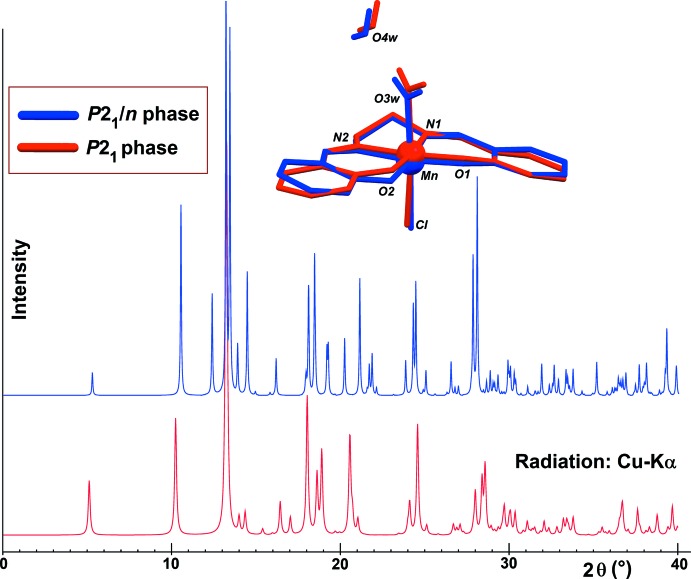
Simulated powder diffraction patterns for the *P*2_1_/*n* form of the title compound (Panja *et al.*, 2003 ▸; blue spectrum) and the *P*2_1_ form (this work; red spectrum). A fit between the mol­ecules constituting the asymmetric units in both phases is also displayed, using the same colour scheme (Macrae *et al.*, 2008 ▸).

The relative position of the lattice water mol­ecule and the complex mol­ecule is very similar in both polymorphs: a fit between the asymmetric units of each phase, carried out using all non-H atoms in the complex, shows that the unique significant differences are for the phenol rings C1–C6 and C11–C16, which are rotated about their σ bonds C7—C6 and C10—C11, by *ca* 6.4 and 13.9°, respectively. However, such a limited change in the conformation of the complex is unlikely to promote the polymorphism. On the other hand, each phase gives a clearly different simulated powder diffraction pattern (Fig. 2 ▸).

The crystal structure reported by Panja *et al.* is based on a primitive monoclinic unit cell with parameters *a* = 6.6470 (2), *b* = 7.3330 (2), *c* = 33.8260 (10) Å and *β* = 95.1650 (17)°. The cell volume *V* is 1642.07 (8) Å^3^, corresponding to a *P*2_1_/*n* structure with four formulas per unit cell. An obvious relation is observed with the parameters of our phase (Table 3 ▸): the cell symmetry is retained, with very similar *a, b* and *β* parameters, while the *c* parameter is almost exactly halved. The resulting cell volume is then *V* = 838.67 (10) Å^3^. Therefore, the unit-cell content is also halved to *Z* = 2, and a marginal difference of 2% for the calculated densities is observed between the two polymorphs. It is worth mentioning that after the data collection was completed, we checked the correctness of the short *c* parameter for the *P*2_1_ polymorph, by re-building the reciprocal space: no extra diffraction spots with indices (*h k l*/2) for a potential supercell are observed in the 0*kl* and *h*0*l* layers. This can be qu­anti­tatively assessed by integrating the collected frames after doubling the *c* parameter: the statistics for intensities over the whole (*hkl*) pattern are then 〈*I*/σ(*I*)〉 = 4.70 if *l* is even (10251 reflections) and 〈*I*/σ(*I*)〉 = 0.16 if *l* is odd (10053 reflections). The previously reported *P*2_1_/*n* polymorph gives much more balanced statistics, 〈*I*/σ(*I*)〉 = 84.74 for *l* = 2*n* and 〈*I*/σ(*I*)〉 = 85.67 for *l* = 2*n* + 1 [given that, apparently, original structure factors are not available anymore for this crystal, intensities *F*_o_^2^ and standard deviations σ(*F*_o_^2^) were generated using the dedicated tool in *PLATON* (Spek, 2009 ▸)]. These statistics support the correctness of the unit cells for both polymorphs.

**Figure 3:**
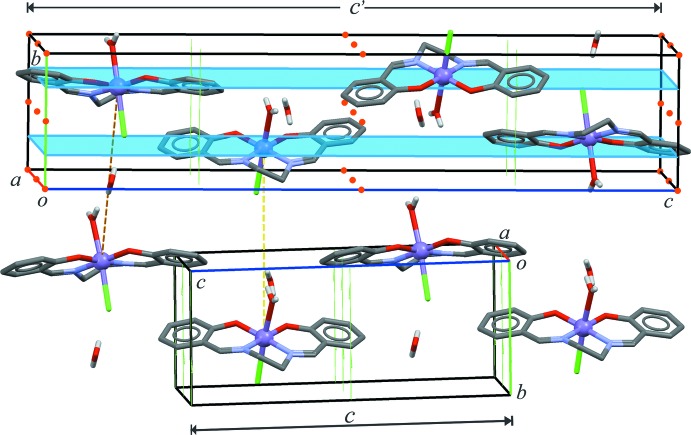
A comparison between the cell content for the *P*2_1_/*n* and *P*2_1_ forms (top and bottom, respectively). Dashed lines relate mol­ecules with identical orientation in both crystals, and symmetry elements are displayed (top: screw axes, glide planes and inversion centres; bottom: screw axes).

A comparison of unit cells shows that mol­ecules related by the screw axis parallel to [010] remain in the same relative orientation (Fig. 3 ▸), including the water mol­ecules. Each pair of mol­ecules is inverted in the *P*2_1_/*n* polymorph, while the lack of a glide plane in the new phase restrains the cell contents to this pair of mol­ecules, which is extended in the crystal through lattice translations. The key point is then that the new phase crystallizes in a non-centrosymmetric space group, *P*2_1_, while doubling the *c* parameter gives a centrosymmetric space group, *P*2_1_/*n.* The presence or absence of an inversion centre affords two phases related by packing polymorphism (Brog *et al.*, 2013 ▸)

**Figure 4:**
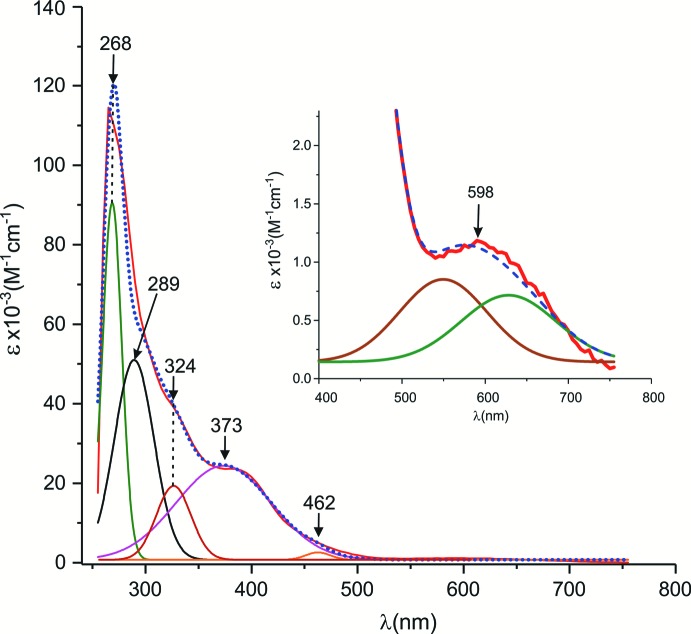
UV–vis spectrum of the title polymorph dissolved in DMSO. The experimental spectrum (red line) is fitted with Gaussian functions for which maxima are indicated. The sum of these Gaussian functions affords the theoretical spectrum (dotted blue line). The visible range of the spectrum is displayed in the inset, using a scale allowing the *d*–*d* transitions to be assessed, fitted with two Gaussian functions, giving a maximum at λ = 598 nm.

The electronic spectrum of the title compound in DMSO shows one band at 264 nm assigned to the ligand π→π^*^ transition, and a broad band at 598 nm, which corresponds to *d*–*d* transitions (Fig. 4 ▸). The *d*–*d* band is satisfactorily fitted with two Gaussian functions (Fig. 4 ▸, inset; OriginLab Corp., 2017 ▸), and can be assigned to the ^5^ *E*_g_→^5^ *T*_2g_ transition, consistent with the distorted octa­hedral ligand field observed for the metal centre in the solid state. If no conformational flexibility is possible for this complex, the polymorphism is then due to different packing structures, rather than geometric modifications.

## Supra­molecular features   

**Figure 5:**
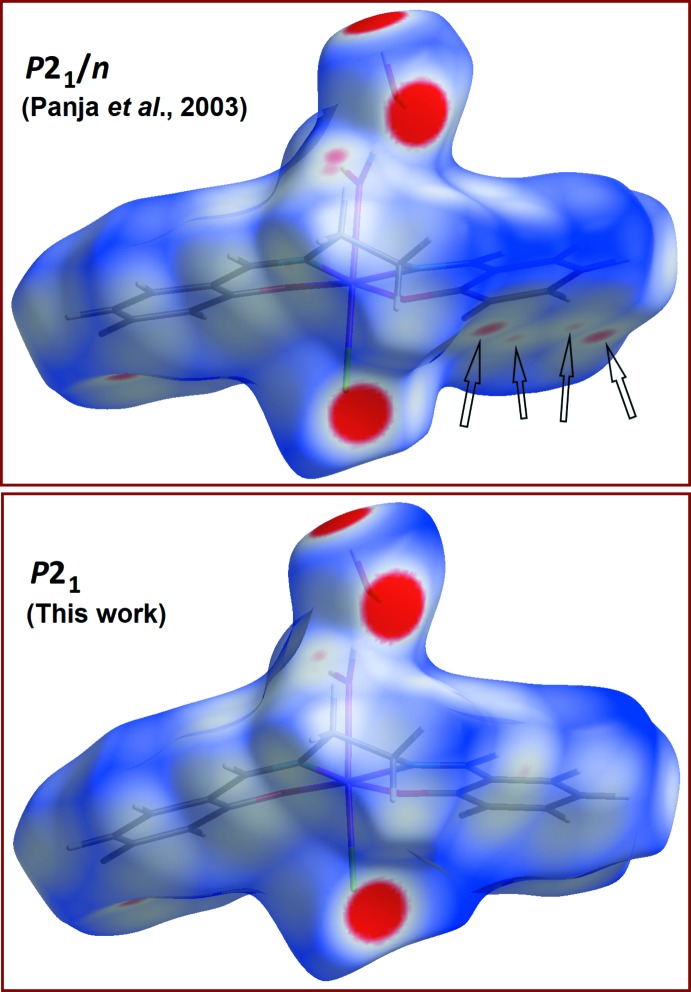
Hirshfeld surfaces mapped over *d_norm_* (−0.15 to 1.20 Å) for the *P*2_1_/*n* polymorph reported in 2003 (Panja *et al.*, 2003 ▸; top) and the novel *P*2_1_ polymorph (bottom). Arrows in the first case indicate regions where inter­molecular distances are shorter than van der Waals contacts, because of π–π inter­actions.

The presence of both a coordinated and a lattice water mol­ecules favours the formation of O—H⋯O hydrogen bonds in the crystal (Table 1 ▸). The coordinated mol­ecule O3*W* serves as donor, forming bonds with the lattice water O4*W* and the chloride atom of a neighbouring complex in position (*x*, *y* − 1, *z*). The lattice mol­ecule O4*W* serves both as donor and acceptor, forming bonds with the chloride and phenolate atom O2 of two symmetry-related complexes. The resulting supra­molecular structure is a 3D framework based essentially on discrete chains extended to large ring motifs. The comparison between the Hirshfeld surfaces for the asymmetric units in the two phases (Fig. 5 ▸; Turner *et al.*, 2017 ▸) is consistent with the observed crystal symmetries and provides some clues about the factor causing the packing polymorphism. For the *P*2_1_/*n* phase, the inversion centre allows the formation of π–π contacts between symmetry-related C11–C16 benzene rings (symmetry code: 2 − *x*, −*y*, 1 − *z*). Such weak inter­actions are reflected in the red spots on the Hirshfeld surface, marked with arrows in Fig. 5 ▸. The main consequence of the absence of an inversion centre in the *P*2_1_ crystal is the removal of these contacts (Fig. 5 ▸, bottom), in connection with the small rotation of 13.9° observed for this part of the Schiff base (see previous section and Fig. 2 ▸).

The crystal structure of the non-hydrated complex has been reported (Martínez *et al.*, 2002 ▸), in space group *P*2_1_, but the packing structure is then modified, since the array of hydrogen bonds is different.

## Database survey   

Retrieving cases of packing polymorphism by mining the Cambridge Structural Database is not a straightforward task, since no dedicated tools have been designed for such a search (CSD, version 5.39, updated May 2018; Groom *et al.*, 2016 ▸). It is thus difficult to estimate whether or not this phenomenon is common. Restraining the search to the symmetry class 2/*m*, we however found some cases very similar to that observed for the title compound, with packing dimorphism in space groups *P*2_1_/*n* and *P*2_1_ (or any alternative settings for these groups), some of which are listed in Table 2 ▸. For each pair, the ratio between the unit-cell volumes for the *P*2_1_/*n* and *P*2_1_ phases is very close to 2, because of the loss of the glide plane and the halving of the cell parameter *c*. Very simple mol­ecules are found, such as glycine (DOLBIR; Arul Asir Abraham *et al.*, 2015 ▸) and also more complex mol­ecules (YURVAI; van den Hende *et al.*, 1995 ▸). Using simulated powder diffraction patterns in order to ensure that a pair of crystal structures forms a genuine case of packing dimorphism, false positive occurrences may also be detected. For example, the reported crystal structures for 4-cyano-4′-ethyl-bipbenyl, referenced KUSVID and KUSVID01 (space groups *P*2_1_/c and *P*2_1_, respectively; Haase *et al.*, 1992 ▸) almost certainly represent the same crystal structure rather than two packing polymorphs resulting from a reversible distortive phase transition, as was reported.

## Synthesis and crystallization   

Equimolar amounts (1 mmol) of MnCl_2_ (0.125 g), salicyl­aldehyde (108 µl) and di­ethyl­enetri­amine (106 µl) in MeOH (5 ml) were placed in a beaker and the mixture was kept under magnetic stirring for 30 minutes at room temperature. As the Schiff base ligand was formed *in situ*, the condensation reaction between the aldehyde and the amine afforded water, which participates as a reagent. The mixture was left at room temperature for one day, filtered, and then cooled to 283 K, affording brown single crystals of the title compound after eight days (51 mg, yield based on Mn: 17%). M.p. 447 K. IR (KBr pellet, cm^−1^): 3436 (O—H), 1610 (C=N), 638 (Mn—O), 460 (Mn—N). The UV–Vis spectrum (Fig. 4 ▸) was measured in a DMSO solution (≃ 1.3×10^−2^ m*M*) using a Cary 50 spectro­photometer (λ_max_/∊, nm/10^−3^ *M* ^−1^cm^−1^): 264/114.5, 598/1.16.

## Refinement   

Crystal data, data collection and structure refinement details are summarized in Table 3 ▸. H atoms for water mol­ecules O3*W* and O4*W* were found in a difference map, and freely refined. Other H atoms were refined as riding on their carrier atoms with C—H = 0.93–0.97 Å and *U*_iso_(H) = 1.2*U*_eq_(C).

## Supplementary Material

Crystal structure: contains datablock(s) I, global. DOI: 10.1107/S2056989018015694/vn2136sup1.cif

Structure factors: contains datablock(s) I. DOI: 10.1107/S2056989018015694/vn2136Isup2.hkl

CCDC reference: 1877342

Additional supporting information:  crystallographic information; 3D view; checkCIF report

## Figures and Tables

**Table 1:** Hydrogen-bond geometry (Å, °)

*D*—H⋯*A*	*D*—H	H⋯*A*	*D*⋯*A*	*D*—H⋯*A*
O3*W*—H31*W*⋯O4*W*	0.81 (5)	2.02 (5)	2.827 (4)	174 (5)
O3*W*—H32*W*⋯Cl^i^	0.80 (6)	2.45 (6)	3.249 (3)	173 (5)
O4*W*—H41*W*⋯O2^ii^	0.83 (6)	2.07 (6)	2.896 (4)	170 (5)
O4*W*—H42*W*⋯Cl^iii^	0.81 (5)	2.41 (5)	3.228 (3)	178 (5)

Symmetry codes: (i) 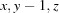; (ii) 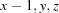; (iii) 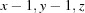.

**Table 2:** Examples of dimorphic crystal structures with packing polymorphism in the symmetry class 2/*m*

CSD references	Space groups	Volumes of unit cells (Å^3^)	Literature reference
CANDUR01, CANDUR02	*P*2_1_/*c*, *P*2_1_	1190, 610	Lutker & Matzger (2010 ▸)
DOLBIR07, DOLBIR08	*P*2_1_/*n*, *P*2_1_	304, 155	Jiang *et al.* (2015 ▸)
GEPSEA01, GEPSEA	*P*2_1_/*c*, *P*2_1_	1108, 554	Konno & Matsushita (2006 ▸)
LIHGAM, LIHGAM01	*P*2_1_/*c*, *P*2_1_	1060, 502	Wang & Fu (2013 ▸)
MIZHOT01, MIZHOT02	*P*2_1_/*c*, *P*2_1_	1472, 738	Sevinçek *et al.* (2011 ▸); Parveen *et al.* (2015 ▸)
NORVEX, NORVEX01	*P*2_1_/*c*, *P*2_1_	1938, 966	Zhang *et al.* (2015 ▸)
SOGUAN20, SOGUAN03	*P*2_1_/*c*, *P*2_1_	1026, 513	Alléaume *et al.* (1976 ▸); Eccles *et al.* (2011 ▸)
YURVAI, YURVAI01	*P*2_1_/*c*, *P*2_1_	4798, 2285	van den Hende *et al.* (1995 ▸); Deacon *et al.* (2014 ▸)

**Table 3:** Experimental details

Crystal data	
Chemical formula	[Mn(C_16_H_14_N_2_O_2_)Cl(H_2_O)]·H_2_O
*M*_r_	392.71
Crystal system, space group	Monoclinic, *P*2_1_
Temperature (K)	298
*a*, *b*, *c* (Å)	6.7083 (4), 7.2414 (5), 17.2768 (13)
β (°)	92.153 (6)
*V* (Å^3^)	838.67 (10)
*Z*	2
Radiation type	Mo *K*α
μ (mm^−1^)	0.97
Crystal size (mm)	0.30 × 0.30 × 0.15

Data collection	
Diffractometer	Agilent Xcalibur Atlas Gemini
Absorption correction	Multi-scan (*CrysAlis PRO*; Agilent, 2013 ▸)
*T*_min_, *T*_max_	0.969, 1.000
No. of measured, independent and observed [*I* > 2σ(*I*)] reflections	10229, 4080, 3647
*R*_int_	0.033
(sin θ/λ)_max_ (Å^−1^)	0.696

Refinement	
*R*[*F* ^2^ > 2σ(*F* ^2^)], *wR*(*F* ^2^), *S*	0.037, 0.072, 1.06
No. of reflections	4080
No. of parameters	229
No. of restraints	1
H-atom treatment	H atoms treated by a mixture of independent and constrained refinement
Δρ_max_, Δρ_min_ (e Å^−3^)	0.40, −0.23
Absolute structure	Flack *x* determined using 1381 quotients [(*I* ^+^)−(*I* ^−^)]/[(*I* ^+^)+(*I* ^−^)] (Parsons *et al.*, 2013 ▸)
Absolute structure parameter	−0.017 (12)

Computer programs: *CrysAlis PRO* (Agilent, 2013 ▸), *SHELXT2018* (Sheldrick, 2015*a* ▸), *SHELXL2018* (Sheldrick, 2015*b* ▸), *Mercury* (Macrae *et al.*, 2008 ▸) and *publCIF* (Westrip, 2010 ▸).

## supplementary crystallographic information

### Crystal data

**Table:**

[Mn(C_16_H_14_N_2_O_2_)Cl(H_2_O)]·H_2_O	*D*_x_ = 1.555 Mg m^−^^3^
*M**_r_* = 392.71	Melting point: 447 K
Monoclinic, *P*2_1_	Mo *K*α radiation, λ = 0.71073 Å
*a* = 6.7083 (4) Å	Cell parameters from 4867 reflections
*b* = 7.2414 (5) Å	θ = 3.7–27.5°
*c* = 17.2768 (13) Å	µ = 0.97 mm^−^^1^
β = 92.153 (6)°	*T* = 298 K
*V* = 838.67 (10) Å^3^	Plate, brown
*Z* = 2	0.30 × 0.30 × 0.15 mm
*F*(000) = 404	

### Data collection

**Table:**

Agilent Xcalibur Atlas Gemini diffractometer	4080 independent reflections
Radiation source: Enhance (Mo) X-ray Source	3647 reflections with *I* > 2σ(*I*)
Graphite monochromator	*R*_int_ = 0.033
ω scans	θ_max_ = 29.7°, θ_min_ = 3.0°
Absorption correction: multi-scan (CrysAlis PRO; Agilent, 2013)	*h* = −9→9
*T*_min_ = 0.969, *T*_max_ = 1.000	*k* = −9→10
10229 measured reflections	*l* = −23→21

### Refinement

**Table:**

Refinement on *F*^2^	Secondary atom site location: difference Fourier map
Least-squares matrix: full	Hydrogen site location: mixed
*R*[*F*^2^ > 2σ(*F*^2^)] = 0.037	H atoms treated by a mixture of independent and constrained refinement
*wR*(*F*^2^) = 0.072	*w* = 1/[σ^2^(*F*_o_^2^) + (0.0269*P*)^2^ + 0.0549*P*] where *P* = (*F*_o_^2^ + 2*F*_c_^2^)/3
*S* = 1.06	(Δ/σ)_max_ < 0.001
4080 reflections	Δρ_max_ = 0.40 e Å^−^^3^
229 parameters	Δρ_min_ = −0.23 e Å^−^^3^
1 restraint	Absolute structure: Flack *x* determined using 1381 quotients [(*I*^+^)-(*I*^-^)]/[(*I*^+^)+(*I*^-^)] (Parsons *et al.*, 2013)
Primary atom site location: structure-invariant direct methods	Absolute structure parameter: −0.017 (12)

### Fractional atomic coordinates and isotropic or equivalent isotropic displacement parameters (Å^2^)

**Table:**

	*x*	*y*	*z*	*U*_iso_*/*U*_eq_	
Mn	0.09362 (6)	0.52766 (6)	0.76613 (3)	0.02725 (13)	
Cl	0.21462 (13)	0.86787 (12)	0.78160 (7)	0.0449 (3)	
O1	0.2621 (3)	0.4339 (3)	0.84472 (14)	0.0314 (5)	
O2	0.2725 (3)	0.4682 (3)	0.68703 (14)	0.0319 (6)	
O3W	−0.0502 (4)	0.2359 (4)	0.7460 (2)	0.0468 (8)	
H31W	−0.167 (8)	0.208 (7)	0.741 (3)	0.070*	
H32W	0.022 (8)	0.150 (8)	0.757 (3)	0.070*	
O4W	−0.4622 (4)	0.1624 (5)	0.7235 (2)	0.0489 (8)	
H41W	−0.541 (8)	0.251 (8)	0.719 (3)	0.073*	
H42W	−0.544 (7)	0.087 (7)	0.737 (3)	0.073*	
N1	−0.1165 (4)	0.5785 (4)	0.84081 (18)	0.0295 (7)	
N2	−0.1111 (4)	0.6231 (4)	0.69079 (18)	0.0314 (7)	
C1	0.2606 (5)	0.4706 (4)	0.9199 (2)	0.0276 (7)	
C2	0.4299 (5)	0.4261 (5)	0.9660 (2)	0.0338 (8)	
H21	0.539320	0.372846	0.943141	0.041*	
C3	0.4374 (6)	0.4598 (5)	1.0446 (2)	0.0415 (9)	
H31	0.552417	0.431406	1.073893	0.050*	
C4	0.2745 (6)	0.5358 (6)	1.0804 (2)	0.0460 (9)	
H41	0.280228	0.559014	1.133357	0.055*	
C5	0.1057 (6)	0.5760 (5)	1.0368 (2)	0.0404 (10)	
H51	−0.004513	0.623686	1.061044	0.048*	
C6	0.0944 (5)	0.5471 (5)	0.95632 (19)	0.0316 (8)	
C7	−0.0886 (5)	0.5890 (4)	0.9141 (2)	0.0324 (8)	
H71	−0.195999	0.627022	0.942692	0.039*	
C8	−0.3150 (5)	0.6098 (5)	0.8028 (2)	0.0380 (9)	
H82	−0.396791	0.684398	0.835810	0.046*	
H81	−0.382002	0.492807	0.793394	0.046*	
C9	−0.2848 (5)	0.7073 (5)	0.7279 (2)	0.0410 (10)	
H91	−0.403077	0.695316	0.694177	0.049*	
H92	−0.260526	0.837639	0.737189	0.049*	
C10	−0.0941 (5)	0.6321 (5)	0.6175 (2)	0.0374 (9)	
H101	−0.202462	0.678747	0.588561	0.045*	
C11	0.0782 (5)	0.5765 (5)	0.5754 (2)	0.0362 (9)	
C12	0.0738 (6)	0.6076 (5)	0.4951 (2)	0.0480 (10)	
H121	−0.040128	0.658706	0.471451	0.058*	
C13	0.2312 (7)	0.5651 (6)	0.4508 (2)	0.0548 (12)	
H131	0.225258	0.587309	0.397749	0.066*	
C14	0.4005 (6)	0.4880 (6)	0.4864 (2)	0.0511 (11)	
H141	0.509161	0.459366	0.456736	0.061*	
C15	0.4104 (5)	0.4534 (5)	0.5645 (2)	0.0381 (9)	
H151	0.524048	0.398346	0.586650	0.046*	
C16	0.2515 (5)	0.4998 (5)	0.61183 (19)	0.0315 (8)	

### Atomic displacement parameters (Å^2^)

**Table:**

	*U*^11^	*U*^22^	*U*^33^	*U*^12^	*U*^13^	*U*^23^
Mn	0.0191 (2)	0.0312 (3)	0.0315 (3)	0.0036 (2)	0.00186 (16)	−0.0002 (2)
Cl	0.0318 (5)	0.0302 (4)	0.0722 (8)	−0.0033 (4)	−0.0032 (4)	0.0006 (4)
O1	0.0250 (12)	0.0383 (13)	0.0308 (14)	0.0071 (10)	0.0006 (10)	−0.0004 (11)
O2	0.0249 (11)	0.0394 (14)	0.0316 (14)	0.0052 (10)	0.0028 (10)	0.0005 (10)
O3W	0.0326 (15)	0.0306 (15)	0.077 (2)	−0.0012 (12)	−0.0035 (15)	0.0004 (14)
O4W	0.0321 (15)	0.0432 (18)	0.072 (2)	0.0047 (13)	0.0084 (14)	0.0092 (15)
N1	0.0179 (13)	0.0290 (16)	0.0417 (19)	0.0024 (10)	0.0022 (12)	−0.0047 (12)
N2	0.0235 (14)	0.0311 (15)	0.0395 (19)	0.0014 (12)	−0.0023 (12)	0.0014 (13)
C1	0.0287 (16)	0.0230 (16)	0.0312 (19)	−0.0042 (13)	0.0027 (14)	0.0035 (13)
C2	0.0294 (18)	0.0333 (19)	0.039 (2)	0.0001 (14)	0.0017 (15)	0.0076 (16)
C3	0.046 (2)	0.040 (2)	0.039 (2)	−0.0069 (17)	−0.0072 (17)	0.0110 (17)
C4	0.067 (2)	0.041 (2)	0.0303 (19)	−0.002 (2)	0.0007 (17)	0.000 (2)
C5	0.050 (2)	0.033 (2)	0.039 (2)	−0.0002 (16)	0.0134 (18)	−0.0011 (15)
C6	0.0311 (16)	0.0283 (18)	0.0356 (19)	−0.0006 (16)	0.0053 (13)	0.0000 (16)
C7	0.0286 (18)	0.0255 (17)	0.044 (2)	−0.0008 (13)	0.0123 (16)	−0.0028 (15)
C8	0.0200 (17)	0.040 (2)	0.054 (3)	0.0034 (15)	0.0047 (16)	−0.0066 (18)
C9	0.0238 (18)	0.038 (2)	0.060 (3)	0.0101 (15)	−0.0049 (17)	−0.0045 (19)
C10	0.0328 (19)	0.0297 (19)	0.049 (3)	−0.0010 (15)	−0.0099 (16)	0.0050 (17)
C11	0.041 (2)	0.031 (2)	0.035 (2)	−0.0024 (15)	−0.0034 (16)	0.0028 (14)
C12	0.062 (3)	0.041 (2)	0.039 (2)	−0.004 (2)	−0.011 (2)	0.0094 (18)
C13	0.085 (3)	0.051 (3)	0.028 (2)	−0.013 (2)	0.003 (2)	0.0035 (18)
C14	0.063 (3)	0.050 (3)	0.042 (2)	−0.011 (2)	0.018 (2)	−0.007 (2)
C15	0.0373 (19)	0.040 (2)	0.037 (2)	−0.0049 (16)	0.0063 (16)	−0.0037 (16)
C16	0.0356 (17)	0.0282 (19)	0.0310 (19)	−0.0063 (16)	0.0034 (14)	0.0001 (16)

### Geometric parameters (Å, º)

**Table:**

Mn—O1	1.862 (2)	C4—H41	0.9300
Mn—O2	1.901 (2)	C5—C6	1.405 (5)
Mn—N1	1.981 (3)	C5—H51	0.9300
Mn—N2	1.981 (3)	C6—C7	1.436 (5)
Mn—O3W	2.343 (3)	C7—H71	0.9300
Mn—Cl	2.6045 (10)	C8—C9	1.496 (5)
O1—C1	1.326 (4)	C8—H82	0.9700
O2—C16	1.322 (4)	C8—H81	0.9700
O3W—H31W	0.81 (5)	C9—H91	0.9700
O3W—H32W	0.80 (6)	C9—H92	0.9700
O4W—H41W	0.83 (6)	C10—C11	1.446 (5)
O4W—H42W	0.81 (5)	C10—H101	0.9300
N1—C7	1.276 (5)	C11—C12	1.405 (5)
N1—C8	1.480 (4)	C11—C16	1.414 (5)
N2—C10	1.277 (5)	C12—C13	1.362 (6)
N2—C9	1.482 (4)	C12—H121	0.9300
C1—C2	1.400 (5)	C13—C14	1.388 (6)
C1—C6	1.414 (4)	C13—H131	0.9300
C2—C3	1.379 (5)	C14—C15	1.372 (5)
C2—H21	0.9300	C14—H141	0.9300
C3—C4	1.389 (5)	C15—C16	1.408 (4)
C3—H31	0.9300	C15—H151	0.9300
C4—C5	1.368 (5)		
			
O1—Mn—O2	93.41 (10)	C6—C5—H51	119.1
O1—Mn—N1	91.15 (11)	C5—C6—C1	119.0 (3)
O2—Mn—N1	173.66 (11)	C5—C6—C7	119.0 (3)
O1—Mn—N2	173.37 (11)	C1—C6—C7	122.0 (3)
O2—Mn—N2	92.71 (11)	N1—C7—C6	125.6 (3)
N1—Mn—N2	82.56 (12)	N1—C7—H71	117.2
O1—Mn—O3W	90.85 (11)	C6—C7—H71	117.2
O2—Mn—O3W	87.51 (11)	N1—C8—C9	107.9 (3)
N1—Mn—O3W	88.01 (11)	N1—C8—H82	110.1
N2—Mn—O3W	86.87 (11)	C9—C8—H82	110.1
O1—Mn—Cl	95.26 (8)	N1—C8—H81	110.1
O2—Mn—Cl	94.89 (7)	C9—C8—H81	110.1
N1—Mn—Cl	89.08 (8)	H82—C8—H81	108.4
N2—Mn—Cl	86.75 (9)	N2—C9—C8	108.3 (3)
O3W—Mn—Cl	173.28 (8)	N2—C9—H91	110.0
C1—O1—Mn	127.9 (2)	C8—C9—H91	110.0
C16—O2—Mn	128.4 (2)	N2—C9—H92	110.0
Mn—O3W—H31W	129 (4)	C8—C9—H92	110.0
Mn—O3W—H32W	115 (4)	H91—C9—H92	108.4
H31W—O3W—H32W	114 (5)	N2—C10—C11	125.9 (3)
H41W—O4W—H42W	96 (4)	N2—C10—H101	117.0
C7—N1—C8	121.8 (3)	C11—C10—H101	117.0
C7—N1—Mn	125.2 (2)	C12—C11—C16	119.3 (3)
C8—N1—Mn	113.0 (2)	C12—C11—C10	117.7 (3)
C10—N2—C9	120.5 (3)	C16—C11—C10	123.0 (3)
C10—N2—Mn	125.7 (2)	C13—C12—C11	122.1 (4)
C9—N2—Mn	113.4 (2)	C13—C12—H121	119.0
O1—C1—C2	118.3 (3)	C11—C12—H121	119.0
O1—C1—C6	123.3 (3)	C12—C13—C14	118.7 (4)
C2—C1—C6	118.3 (3)	C12—C13—H131	120.6
C3—C2—C1	121.2 (3)	C14—C13—H131	120.6
C3—C2—H21	119.4	C15—C14—C13	121.2 (4)
C1—C2—H21	119.4	C15—C14—H141	119.4
C2—C3—C4	120.6 (3)	C13—C14—H141	119.4
C2—C3—H31	119.7	C14—C15—C16	121.2 (4)
C4—C3—H31	119.7	C14—C15—H151	119.4
C5—C4—C3	119.2 (3)	C16—C15—H151	119.4
C5—C4—H41	120.4	O2—C16—C15	118.3 (3)
C3—C4—H41	120.4	O2—C16—C11	124.1 (3)
C4—C5—C6	121.7 (3)	C15—C16—C11	117.6 (3)
C4—C5—H51	119.1		
			
O2—Mn—O1—C1	160.2 (2)	Mn—N1—C8—C9	−34.8 (3)
N1—Mn—O1—C1	−24.2 (2)	C10—N2—C9—C8	155.3 (3)
O3W—Mn—O1—C1	−112.2 (2)	Mn—N2—C9—C8	−31.2 (4)
Cl—Mn—O1—C1	65.0 (2)	N1—C8—C9—N2	41.5 (4)
Mn—O1—C1—C2	−163.0 (2)	C9—N2—C10—C11	171.8 (3)
Mn—O1—C1—C6	18.9 (4)	Mn—N2—C10—C11	−0.7 (5)
O1—C1—C2—C3	−179.7 (3)	N2—C10—C11—C12	−176.2 (3)
C6—C1—C2—C3	−1.5 (5)	N2—C10—C11—C16	1.4 (6)
C1—C2—C3—C4	1.3 (5)	C16—C11—C12—C13	0.4 (6)
C2—C3—C4—C5	0.4 (6)	C10—C11—C12—C13	178.0 (4)
C3—C4—C5—C6	−1.8 (6)	C11—C12—C13—C14	0.3 (6)
C4—C5—C6—C1	1.5 (5)	C12—C13—C14—C15	0.5 (6)
C4—C5—C6—C7	178.8 (4)	C13—C14—C15—C16	−1.9 (6)
O1—C1—C6—C5	178.2 (3)	Mn—O2—C16—C15	176.1 (2)
C2—C1—C6—C5	0.2 (5)	Mn—O2—C16—C11	−4.3 (5)
O1—C1—C6—C7	0.9 (5)	C14—C15—C16—O2	−177.9 (3)
C2—C1—C6—C7	−177.1 (3)	C14—C15—C16—C11	2.5 (5)
C8—N1—C7—C6	175.0 (3)	C12—C11—C16—O2	178.7 (3)
Mn—N1—C7—C6	−6.9 (5)	C10—C11—C16—O2	1.2 (5)
C5—C6—C7—N1	176.2 (3)	C12—C11—C16—C15	−1.7 (5)
C1—C6—C7—N1	−6.5 (5)	C10—C11—C16—C15	−179.2 (3)
C7—N1—C8—C9	143.5 (3)		

### Hydrogen-bond geometry (Å, º)

**Table:**

*D*—H···*A*	*D*—H	H···*A*	*D*···*A*	*D*—H···*A*
O3*W*—H31*W*···O4*W*	0.81 (5)	2.02 (5)	2.827 (4)	174 (5)
O3*W*—H32*W*···Cl^i^	0.80 (6)	2.45 (6)	3.249 (3)	173 (5)
O4*W*—H41*W*···O2^ii^	0.83 (6)	2.07 (6)	2.896 (4)	170 (5)
O4*W*—H42*W*···Cl^iii^	0.81 (5)	2.41 (5)	3.228 (3)	178 (5)
C8—H81···O1^ii^	0.97	2.61	3.217 (4)	121

Symmetry codes: (i) *x*, *y*−1, *z*; (ii) *x*−1, *y*, *z*; (iii) *x*−1, *y*−1, *z*.
